# Association between human leukocyte antigen and immunosuppressive treatment outcomes in Chinese patients with aplastic anemia

**DOI:** 10.3389/fimmu.2023.1056381

**Published:** 2023-01-30

**Authors:** Lingyun Chen, Meili Ge, Jiali Huo, Xiang Ren, Yingqi Shao, Xingxin Li, Jinbo Huang, Min Wang, Neng Nie, Jing Zhang, Jin Peng, Yizhou Zheng

**Affiliations:** State Key Laboratory of Experimental Hematology, National Clinical Research Center for Blood Diseases, Haihe Laboratory of Cell Ecosystem, Institute of Hematology & Blood Diseases Hospital, Chinese Academy of Medical Sciences & Peking Union Medical College, Tianjin, China

**Keywords:** aplastic anemia, HLA, immunosuppressive therapy, therapy response, clonal evolution

## Abstract

**Background:**

Activated cytotoxic T cells (CTLs) recognize the auto-antigens presented on hematopoietic stem/progenitor cells (HSPCs) through class I human leukocyte antigen (HLA) molecules and play an important role in the immune pathogenesis of aplastic anemia (AA). Previous reports demonstrated that HLA was related to the disease susceptibility and response to immunosuppressive therapy (IST) in AA patients. Recent studies have indicated that specific HLA allele deletions, which helped AA patients to evade CTL-driven autoimmune responses and escape from immune surveillance, may lead to high-risk clonal evolution. Therefore, HLA genotyping has a particular predictive value for the response to IST and the risk of clonal evolution. However, there are limited studies on this topic in the Chinese population.

**Methods:**

To explore the value of HLA genotyping in Chinese patients with AA, 95 AA patients treated with IST were retrospectively investigated.

**Results:**

The alleles HLA-B*15:18 and HLA-C*04:01 were associated with a superior long-term response to IST (P = 0.025; P = 0.027, respectively), while the allele HLA-B*40:01 indicated an inferior result (P = 0.02). The allele HLA-A*01:01 and HLA-B*54:01 were associated with high-risk clonal evolution (P = 0.032; P = 0.01, respectively), and the former had a higher frequency in very severe AA (VSAA) patients than that in severe AA (SAA) patients (12.7% vs 0%, P = 0.02). The HLA-DQ*03:03 and HLA-DR*09:01 alleles were associated with high-risk clonal evolution and poor long-term survival in patients aged ≥40 years. Such patients may be recommended for early allogeneic hematopoietic stem cell transplantation rather than the routine IST treatment.

**Conclusion:**

HLA genotype has crucial value in predicting the outcome of IST and long-term survival in AA patients, and thus may assist an individualized treatment strategy.

## 1 Introduction

Acquired aplastic anemia (AA) is a rare disease characterized by peripheral blood pancytopenia and bone marrow hematopoietic failure. At present, the activated T cell-mediated destruction of hematopoietic stem/progenitor cells (HSPCs) is the widely-recognized pathogenesis of AA (1, 2). Therefore, immunosuppressive therapy (IST) is recommended as the first-line treatment choice for severe AA (SAA) patients lacking human leukocyte antigen (HLA) -matched sibling donors and older ones (3). However, up to 30% of SAA patients failed to respond to IST (3). Moreover, in a long-term follow-up, 9% of patients relapsed, and 11% developed clonal evolution (4). Therefore, it is critical to early identify which patients can benefit from IST.

HLA is closely related to the function of the immune system. Early studies of AA mainly focused on HLA- class II genes (5), especially HLA-DR. Several related studies have shown that the frequency of HLA-DR15 and HLA-DR2 alleles in AA patients is significantly higher than that in healthy controls (6–9), and patients with genotype HLA-DR15 or HLA-DR2 respond well to IST (7–10). Katagiri (11) revealed the relationship between HLA-class I alleles and AA. They found that autoimmunity to HSPCs was mediated by specific HLA-class I molecules presenting antigens to cytotoxic T lymphocytes (CTLs), while the loss of heterozygosity on the short arm of chromosome 6 (6pLOH) was the process of immune escape, and the loss of HLA alleles in 6pLOH were obviously biased towards specific HLA types. Again, Babushok (12) showed the HLA class I-driven autoimmunity in AA. They discovered that AA patients who inherited targeted HLA alleles had a more severe course of the disease and predisposition to clonal evolution, regardless of the status of HLA mutations, which was consistent with the findings of Zaimoku (13). Therefore, HLA genotyping probably has a particular predictive value for the response to IST and the risk of clonal evolution. However, the polymorphisms of HLA alleles are distinct in different ethnic groups (6, 14–18), and there are few related studies in the Chinese population. This study aimed to investigate the association of HLA-class I and HLA-class II alleles with the response to IST and the risk of clonal evolution in Chinese patients.

## 2 Methods

### 2.1 Patients

A total of 202 AA patients who underwent HLA typing in Blood Diseases Hospital, Chinese Academy of Medical Sciences & Peking Union Medical College from January 2012 to December 2020 were retrospectively analyzed, and 95 of these patients received IST treatment. AA was diagnosed by bone marrow biopsy and peripheral blood count based on the criteria of the International Agranulocytosis and Aplastic Anemia Study Group (19). Disease severity was assessed according to Camitta criteria (20). If the patient had (1): marked myeloid hypoplasia (degree of hyperplasia <25% of normal) or non-severe hypoplasia (degree of hyperplasia of 25-50% of normal cells and residual hematopoietic cells <30%) and (2) At least two of the following three peripheral blood counts were met: absolute neutrophil count (ANC)<0.5×10^9^/L; absolute reticulocyte count (ARC)<20×10^9^/L; platelet (PLT)<20×10^9^/L can be diagnosed as SAA. Patients with ANC <0.2×10^9^/L can be diagnosed with very severe AA (VSAA). There were no patients with congenital AA in this study, and all patients had negative Ham test results and no clinical or laboratory-related signs of hemolysis.

Ninety-five patients were treated with cyclosporine (CsA) and Rabbit anti-thymocyte globulin (r-ATG) or Porcine anti-lymphocyte globulin(p-ALG). rATG was administered at a dose of 3~4mg/kg per day for five days. p-ALG was administered at a dose of 20~30mg/kg per day for five days. CsA was administered every 12 hours with a dose of 3~5mg/kg/d, which was adjusted according to serum levels. The response to IST was evaluated according to the following criteria. Complete response (CR) was defined as hemoglobin(HGB)>120g/L (male) or 110g/L (female), ANC>1.5×10^9^/L, PLT>100×10^9^/L; Partial response (PR) was defined as an increase in peripheral blood cell counts compared with baseline levels, and independent of blood product transfusions for at least three consecutive months, no longer meeting the diagnostic criteria for SAA; Non-response (NR) was defined as peripheral blood counts that still met diagnostic criteria for SAA and/or the patient remained transfusion-dependent. Response rates included CR and PR, and patients who died within 3 months after IST were recorded as NR. High-risk clonal evolution was defined as acquiring chromosome 7 abnormalities, complex cytogenetics, or transformation to myelodysplastic syndrome (MDS) or acute myeloid leukemia (AML). The deadline for follow-up of all patients was December 2021, and follow-up was discontinued when the patient died or underwent allogeneic hematopoietic stem cell transplantation. Overall survival (OS) time was defined as the time from the first day of IST treatment to the last follow-up or when the patient died of any cause.

### 2.2 HLA typing

A peripheral blood sample, anti-coagulated with ethylenediamine tetraacetic acid (EDTA)-K2 was obtained, and HLA typing was undertaken using next generation sequencing (NGS) technology. The variant was detected by NGSgo HLA typing kit (GenDx, Utrecht, The Netherlands) on the Illumina MiniSeq system platform (lllumina, San Diego, CA, USA). Sequences were analyzed using NGSenqine analysis software (version 2.23; GenDX, lnc). HLA allele typing was carried out according to the serological method and named according to the international standard. The first two digits after the * symbol refer to the corresponding serological specificity of the allele, and the last two digits represent the sequence number of the allele.

### 2.3 Gene sequencing

Deoxyribonucleic acid (DNA) was extracted from bone marrow samples, and a polymerase chain reaction (PCR) machine was used to amplify DNA. DNA amplification product was quantified by agarose gel electrophoresis and Nanodrop (Thermo). Libraries were prepared using Illumina standard protocol. The single-stranded library DNA fragments enter the flow cell of the Illumina sequencing platform and were captured with a 114 Gene Panel using biotinylated oligo-probes (MyGenostics GenCap Enrichment technologies). Library fragments were used as templates for DNA replication. After the replication was completed, the chain was unchained, and the library fragments were washed away. Illumina utilizes a unique “bridged” amplification reaction that occurs on the surface of the flow cell. A flow cell containing millions of unique clusters is loaded into the HiSeq 2000 for automated cycles of extension and imaging. Then Melted and washed out the synthesized part of the sequencing, added the clustering primer Read2, and read the other end sequence from the opposite direction.

### 2.4 Statistical analysis

SPSS 22.0 software was used for statistical analysis. The Shapiro-Wilk method was used to test whether the continuous variables satisfy the normal distribution. The differences in categorical variables between the groups carrying a certain HLA allele and those not carrying a certain HLA allele were compared by Pearson’s χ^2^ analysis or Fisher’s exact test. The differences in non-normally distributed continuous variables were compared by the Mann-Whitney U test. Survival analysis was performed by the Kaplan-Meier method, and Log-rank was used to compare the significance of the differences. *P*<0.05 for the two-sided test was considered statistically significant.

## 3 Results

### 3.1 Association between HLA genotype frequencies and patient clinical parameters

Two hundred and two patients undergoing HLA typing were included in the study of differences in clinical parameters at initial diagnosis, and summarized in **Supplemental Table 1**. Among them, HLA-A*01:01 and HLA-DR15:01 alleles were related to the disease severity. In VSAA patients, the proportion of patients with genotypes HLA-A*01:01, HLA-DQ*03:03 and HLA-DR*09:01 were significantly higher than that in SAA patients (13.3% vs 5.2%, *P* = 0.047, OR = 0.353; 48.6% vs 27.8%, *P* = 0.002, OR = 0.408; 44.8% vs 27.8%, *P* = 0.013, OR = 0.476; **Supplemental Table 1**). In contrast, there were 24 (22.9%) VSAA patients with the HLA-DR15:01 genotype, which was significantly lower than 40 (41.2%) of SAA patients (*P* = 0.005, OR = 2.368, **Supplemental Table 1**). The HLA-C*08:01 and HLA-DR*15:01 alleles showed gender differences. There were 14 (11.6%) and 31 (25.6%) male patients with the two genotypes, while 19 (23.5%) and 33 (40.1%) female patients with these two genotypes. The age of disease onset was associated with 6 HLA genotypes. The age of onset was late for patients carrying HLA-B*13:01 and HLA-DQ*03:01 and early when carrying HLA-DQ*03:03, HLA-DQ*06:01, HLA-DR*08:03, and HLA-DR*09:01. The alleles HLA-DQ*03:03 and HLA-DR*09:01 were associated with lower pretreatment ANC values (0.17 [0-1.12] ×10^9^/L vs 0.29 [0-1.31] ×10^9^/L, *P* = 0.002; 0.17[0-1.12] ×10^9^/L vs 0.28[0-1.31] ×10^9^/L, *P* = 0.038; **Supplemental Table 1**), and HLA-DQ*03:01was associated with higher ANC values (0.30 [0-1.31] ×10^9^/L vs 0.20 [0-0.96] ×10^9^/L, *P* = 0.04; **Supplemental Table 1**). Besides, the allele HLA-B*15:01 was associated with lower pretreatment platelet values (5[1-17] ×10^9^/L vs 10[1-46] ×10^9^/L, *P* = 0.001; **Supplemental Table 1**).

### 3.2 Association of HLA genotype frequencies with the response to IST

Ninety-five patients received IST therapy. There were 49 males and 46 females, including 40 patients with SAA and 55 patients with VSAA. The median age was 22 years (range 5-60 years). The baseline characteristics of the 95 patients were summarized in **Table 1**.

**Table 1:** Baseline clinical characteristics of patients treated with IST.

	Value
Sex	95(100%)
Male	49(51.6%)
Female	46(48.4%)
age, y	22(15-32)
<18y	29(30.5%)
≥18 and <40y	49(51.6%)
≥40y	17(17.9%)
Severity of disease	95(100%)
SAA	40(42.1%)
VSAA	55(57.9%)
Pretreatment blood values	
Neutrophil count	0.18(0.07-0.46)
Lymphocyte count	1.31(0.69-2.0)
Reticulocyte count	9.8(5.3-16.8)
Platelet count	9(5-13)
Treatment	
Rabbit-ATG+ CsA	42(44.2%)
Porcine-ALG+ CsA	53(55.8%)

Values are n (%) or median (IQR). SAA, severe aplastic anemia; VSAA, very severe aplastic anemia; ATG, anti-thymocyte globulin; ALG, anti-lymphocyte globulin; CsA, cyclosporine

At the end of the follow-up, a total of 60 (63.2%) patients responded to IST, including 45 (47.4%) CR and 15 (15.8%) PR. The relationship between long-term response after IST and HLA genotype frequency was shown in **Table 2**. Alleles HLA-B*15:18, HLA-B*40:01, and HLA-C*04:01 alleles were associated with the long-term response to IST. In the response group, the frequency of the HLA-B*15:18 genotype was significantly higher than that in the unresponsive group (13.3% vs 0%, *P* = 0.025, **Table 2**). Similarly, the presence of HLA-C*04:01 allele was more frequent in the response group than that in the unresponsive group. (20.0% vs 2.9%, *P* = 0.027, **Table 2**). However, in contrast, the genotype frequency of HLA-B*40:01 was obviously lower in the responsive group (10% vs 28.6%, *P* = 0.02, **Table 2**). The rest HLA alleles were presented in the **Supplemental Table 2**.

**Table 2:** Phenotype of frequencies of HLA alleles according to Long-term response to immunosuppressive therapy or incidence of high-risk clonal evolution.

HLA allele	Total	Long-term response to immunosuppressive therapy			High-risk clonal evolution		
		Response (n=60) n (%)^a^	Nonresponse (n=35) n (%)^b^	P value	Yes (n=11) n (%)^c^	No (n=84) n (%)^d^	P value
HLA-A*01:01	7	2(3.3)	5(14.3)	0.096	3(27.2)	4(4.8)	0.032
HLA-B*15:18	8	8(13.3)	0(0)	0.025	0(0)	8(9.5)	0.590
HLA-B*40:01	16	6(10.0)	10(28.6)	0.020	4(36.4)	12(14.3)	0.086
HLA-B*54:01	5	1(1.7)	4(11.4)	0.060	3(27.2)	2(2.4)	0.010
HLA-C*04:01	13	12(20.0)	1(2.9)	0.027	0(0)	13(15.5)	0.351

^a^% of total response patients; ^b^% of total nonresponse patients; ^c^% of total developed high-risk clonal evolution patients; ^d^% of total undeveloped high-risk clonal evolution patients.

We further assessed the relationship between HLA genotype frequency and treatment response at 3, 6, and 12 months after IST treatment, as shown in **Table 3**. The response to IST was 43.2% (1.1% CR and 42.1% PR) at 3 months; 53.7% (8.4% CR and 45.3% PR) at 6 months; 63.2% (30.5% CR and 33.7% PR) at 12 months. Two HLA genotypes were associated with a poor response to IST. Patients with the HLA-A*02:07 allele responded poorly at 3 and 6 months after IST (82.4% vs 17.6%, *P* = 0.019; 70.6% vs 29.4%, *P* = 0.027, respectively; **Table 3**). However, the difference in response disappeared when it comes to 12 months (47.1% vs 52.9%, *P* = 0.285; **Table 3**). The HLA-B*40:01 genotype predicted an inferior response at 6 and 12 months after IST (753% vs 25%, *P* = 0.012; 62.5% vs 37.5%, *P* = 0.015, respectively; **Table 3**). Two HLA alleles were correlated with a good response to IST. The frequency of HLA-B*15:18 genotype in the response group was higher than that in the unresponsive group at 3 months, 6 months and 12 months (62.5% vs 37.5%, *P* = 0.285; 87.5% vs 12.5%, *P* = 0.065; 100% vs 0%, *P* = 0.019, respectively; **Table 3**). But the difference was only significant at 12 months. Similarly, the frequency of the HLA-A*31:01 genotype was higher in the response group at both 3 and 6 months after IST while with no significant difference (75% vs 25%, *P* = 0.072; 87.5% vs 12.5%, *P* = 0.065, respectively; **Table 3**). At 12 months, all 8 patients with HLA-A*31:01 genotype responded to IST (100% vs 0%, *P* = 0.019; **Table 3**). However, one of these patients developed MDS at 32 months and died 41 months after IST.

**Table 3:** Phenotype of frequencies of HLA alleles according to response to immunosuppressive therapy.

HLA allele	Total	Response n(%)	Nonresponse n(%)	P value
3 months after IST		n=41	n=54	
HLA-A*02:07	17	3(17.6)	14(82.4)	**0.019**
HLA-A*31:01	8	6(75)	2(25)	**0.072**
HLA-B*15:18	8	5(62.5)	3(37.5)	**0.285**
HLA-B*40:01	16	5(31.3)	11(68.8)	**0.292**
HLA-C*04:01	13	6(46.2)	7(53.8)	**0.814**
6 months after IST		n=51	n=44	
HLA-A*02:07	17	5(29.4)	12(70.6)	**0.027**
HLA-A*31:01	8	7(87.5)	1(12.5)	0.065
HLA-B*15:18	8	7(87.5)	1(12.5)	0.065
HLA-B*40:01	16	4(25)	12(75)	**0.012**
HLA-C*04:01	13	10(76.9)	3(23.1)	**0.071**
12 months after IST		n=61	n=34	
HLA-A*02:07	17	9(52.9)	8(47.1)	**0.285**
HLA-A*31:01	8	8(100)	0(0)	**0.047**
HLA-B*15:18	8	8(100)	0(0)	**0.047**
HLA-B*40:01	16	6(37.5)	10(62.5)	**0.015**
HLA-C*04:01	13	11(84.6)	2(15.4)	**0.126**

% of response/nonresponse in patients carrying the HLA alleles.

### 3.3 Association of HLA genotype frequencies with high-risk clonal evolution

**Figure 1:**
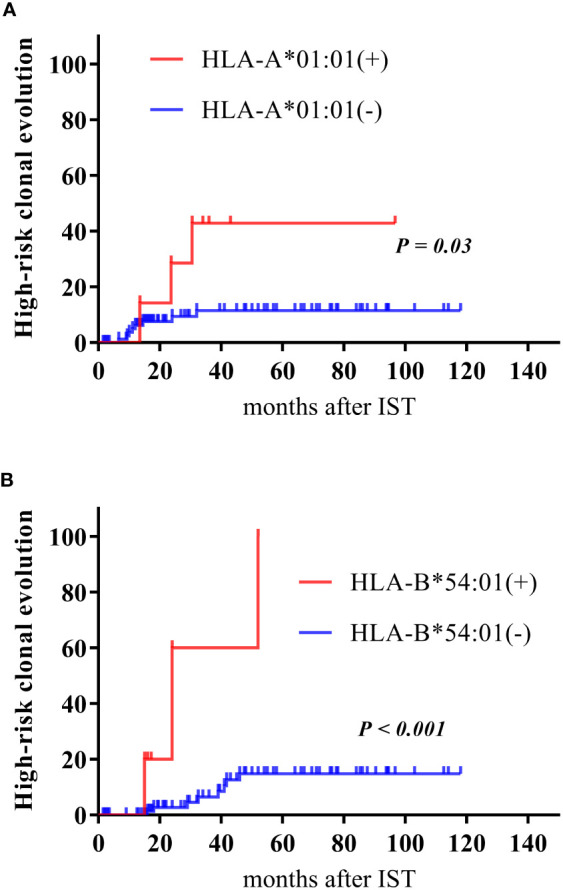
Cumulative incidence of high-risk clonal evolution. Cumulative incidence of high-risk clonal evolution according to patients carrying or not carrying the HLA-A*01:01 allele **(A)**, and the HLA-B*54:01 **(B)**.

A total of 11 patients (11.6%) developed high-risk clonal evolution. The occurrence of high-risk clonal evolution was associated with HLA-A*01:01 and HLA-B*54:01 genotypes. Among the 11 patients with high-risk clonal evolution, 3 patients (27.3%) had genotypes of HLA-A*01:01 and HLA-B*54:01, which were much higher than those without clonal evolution (4.8%, *P* = 0.032 and 2.4%, *P* = 0.01, respectively; **Table 2**). The rest HLA alleles were presented in the **Supplemental Table 2**. Cumulative incidence of high-risk clonal evolution showed the same results (**Figures 1A, B**).

### 3.4 Subgroup analysis

Since age and disease severity were independent predictors of response to IST and clonal evolution in AA patients (21–24), patients were further subdivided into three groups according to age (<18 years old, ≥18 and <40 years old, ≥40 years old) and two groups (VSAA and SAA) according to the disease severity.

In patients aged≥40 years old, a total of 5 patients carried the HLA-DQ*03:03 allele, and they carried the HLA-DR*09:01 allele at the same time. And such genotypes were associated with poor long-term response to IST (0% vs 100%, *P* = 0.029) and high incidence of high-risk clonal evolution (60% vs 40%, *P* = 0.015). In the 18≤n<40-year-old group, the incidence of clonal evolution in patients with the HLA-B*54:01 allele was significantly higher than that of non-carriers (100% vs 8.7%, *P*=0.002). In the <18 years old group, patients had a higher long-term response rate (75.9%), a lower frequency of clonal evolution (3.4%), and no associated risk HLA alleles.

In the SAA group, patients with the HLA-A*02:07 genotype had significantly lower long-term response rates and higher incidence of clonal evolution than those without the HLA-A*02:07 genotype (33.3% vs 79.4%, *P* = 0.039; 50% vs 5.9%, *P* = 0.018; **Table 4**). In addition, the HLA-B*54:01 allele was associated with high-risk clonal evolution (*P* = 0.036; **Table 4**). In the VSAA group, the long-term response of patients carrying the HLA-B*15:18 allele was better than the patients without this allele (100% vs 51%, *P* = 0.03; **Table 4**, while patients with HLA-B*40:01 allele achieved inferior long-term response than those without HLA-B*40:01 allele (14.3% vs 62.5%, *P* = 0.035; **Table 4**). Moreover, HLA-A*01:01 allele was associated with high-risk clonal evolution (*P* = 0.021; **Table 4**).

For patients aged 18-40 years and diagnosed with SAA, HLA-C*01:02 alleles were associated with poor long-term response to IST (*P* = 0.031; **Table 4**), and HLA-B*54:01 alleles were associated with a high risk of clonal evolution (*P* = 0.014; **Table 4**). While for patients aged 18-40 years with a diagnosis of VSAA, HLA-B*40:01 alleles were associated with poor long-term response to IST (*P* = 0.035; **Table 4**), and HLA-A*01:01 alleles were associated with the predisposition to high-risk clonal evolution (*P* = 0.045; **Table 4**).

**Table 4:** Association of HLA alleles with Long-term response to immunosuppressive therapy or incidence of high-risk clonal evolution in aplastic anemia patients.

HLA allele	Long-term response rate(%)			High-risk clonal evolution rate(%)		
	Allele(+) patients	Allele(-) patients	P value	Allele(+) patients	Allele(-) patients	P value
SAA						
HLA-A*02:07	2(33.3)	27(79.4)	0.039	3(50)	2(5.9)	0.018
HLA-B*54:01	1(33.3)	18(75.7)	0.178	2(66.7)	3(8.1)	0.036
VSAA						
HLA-A*01:01	2(28.6)	29(60.4)	0.220	3(42.9)	3(6.3)	0.022
HLA-B*15:18	6(100)	25(51)	0.030	0(0)	6(12.2)	1.000
HLA-B*40:01	1(14.3)	30(62.5)	0.035	1(14.3)	5(10.4)	0.577
Age(18≤n<40y)						
HLA-B*54:01	0(0)	30(65.2)	0.053	3(100)	4(8.7)	0.002
Age(≥40y)						
HLA-DQ*03:03	0(0)	8(66.7)	0.029	3(60)	0(0)	0.015
HLA-DR*09:01	0(0)	8(66.7)	0.029	3(60)	0(0)	0.015
Age(18≤n<40y) and SAA						
HLA-B*54:01	0(0)	15(78.9)	0.071	2(100)	1(5.3)	0.014
HLA-C*01:02	2(33.3)	13(86.7)	0.031	2(33.3)	1(6.7)	0.184
Age(18≤n<40y) and VSAA						
HLA-A*01:01	0(0)	15(60)	0.087	2(66.7)	2(8)	0.045
HLA-B*40:01	0(0)	15(62.5)	0.035	1(25)	3(12.5)	0.481

### 3.5 Survival

**Figure 2:**
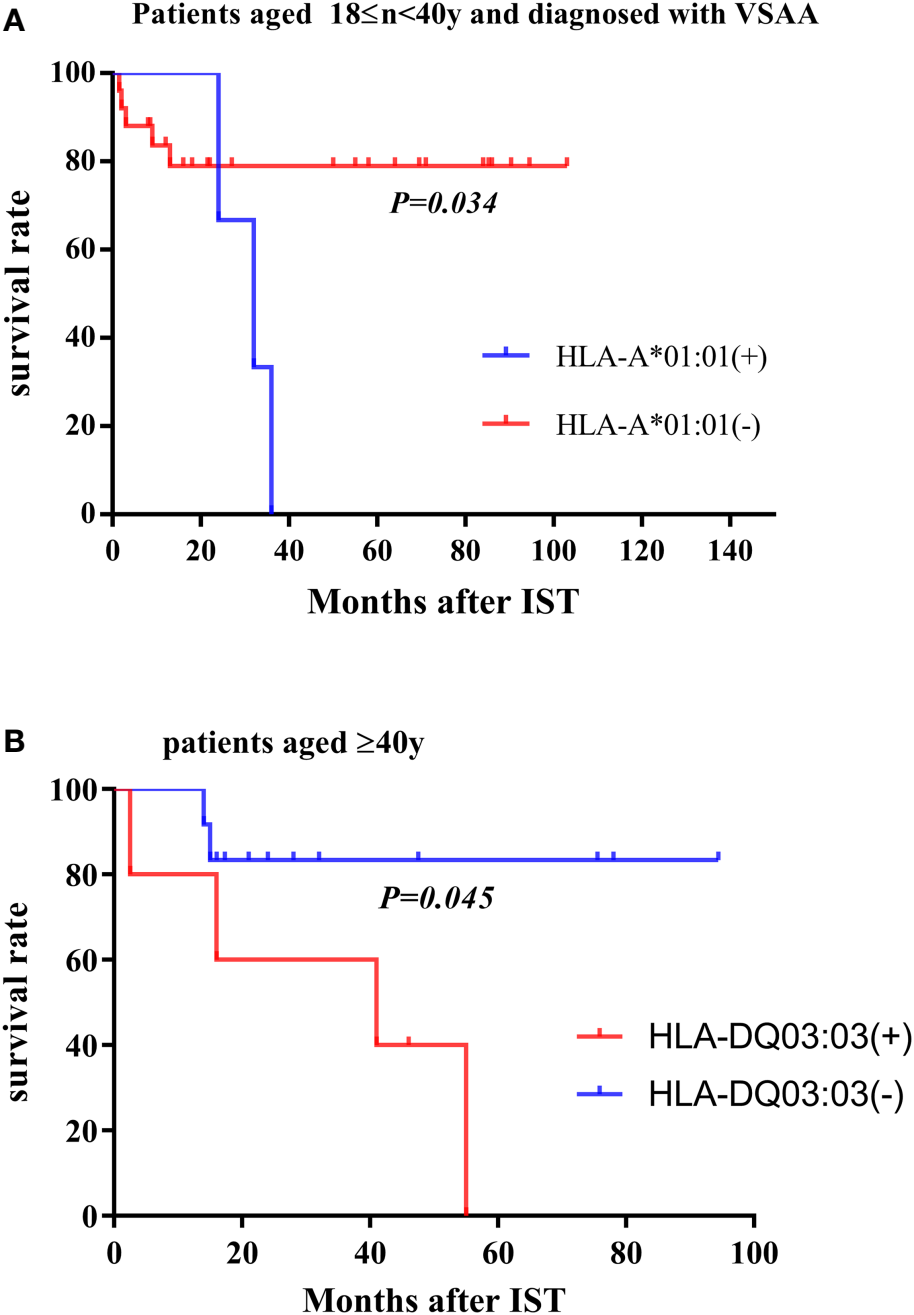
Survival rate in subgroups. **(A)** In the population aged 18≤n<40y and diagnosed with VSAA, the overall survival rate of patients carrying the HLA-A*01:01 allele and other patients not carrying the HLA allele. **(B)** In patients >40 years of age, overall survival of patients carrying the HLA-DQ*03:03 allele (or HLA-DR*09:01 allele) and other patients not carrying the HLA allele.

A total of 20 (21.1%) of 95 patients died. The median OS time in this study was 34 months (range 1.5-116.5 months). The previously described HLA genotypes associated with response to IST and clonal evolution were further analyzed in the survival. The results showed that for VSAA patients aged ≥18 years and <40 years old, HLA-A*01:01 allele was associated with poor survival (*P* = 0.034, **Figure 2A**). For patients aged >40 years old, both the HLA-DQ*03:03 and HLA-DR*09:01 alleles were associated with lower survival rates (*P* = 0.045, **Figure 2B**).

### 3.6 Association of HLA genotype frequencies with somatic mutations

**Figure 3:**
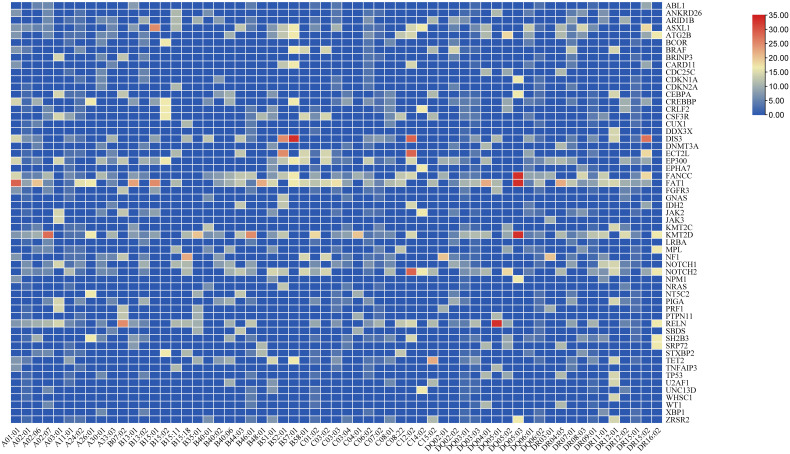
Somatic mutations related to hematological diseases. HLA alleles associated with somatic mutations. Each grid represents the frequency of the somatic mutation in patients carrying the HLA allele.

Somatic mutations were detected in 128 patients at initial diagnosis. FAT1 was the most common somatic mutation in AA, with 12 (9.4%) patients mutated. Following FAT1 was KMT2D, mutated in 10 (7.8%) patients. RELN, NOTCH2, FANCC, and DIS3 were also prone to mutations in AA, with frequencies of 6.3%, 5.5%, 5.5%, and 4.7%, respectively. The mean variant allele frequencies (VAF) of FAT1 and DIS3 were 49.6 ± 2.9% and 48.5 ± 3.1%. The median VAF of KMT2D, RELN, NOTCH2 and FANCC were 48.2%(45.2-49.9%),48.5%(44.9-49.4%),49.3%(46.7-50.4%), and 48.5(46.0-50.5%), respectively. **Figure 3** shows the frequency of AA-related somatic mutations in patients carrying the HLA alleles. As shown in the figure, patients with HLA-B*57:01 have a high frequency of DIS3 mutation, and patients with genotype HLA-DQ*05:01 have a high frequency of RELN mutation. And the frequency of FANCC, FAT1, and KMT2D mutation was high in patients with the HLA-DQ*05:03 genotype.

We further compared the relationship between somatic mutations and HLA alleles associated with response to IST and clonal evolution. Patients carrying the HLA-A*01:01 allele had a higher frequency of FAT1 mutation than non-carriers (*P* = 0.027). Two patients with KMT2C mutations were both genotypes of HLA-B*40:01 (*P* = 0.015). The KMT2D mutation was more frequent in patients with HLA-A*02:07 than those without HLA-A*02:07 (22.2% vs 5.5%, *P* = 0.034). Besides, the frequency of CUX1 mutation was higher in patients with HLA-B*15:18 than in patients without HLA-B*15:18 (*P* = 0.004).

**Figure 4:**
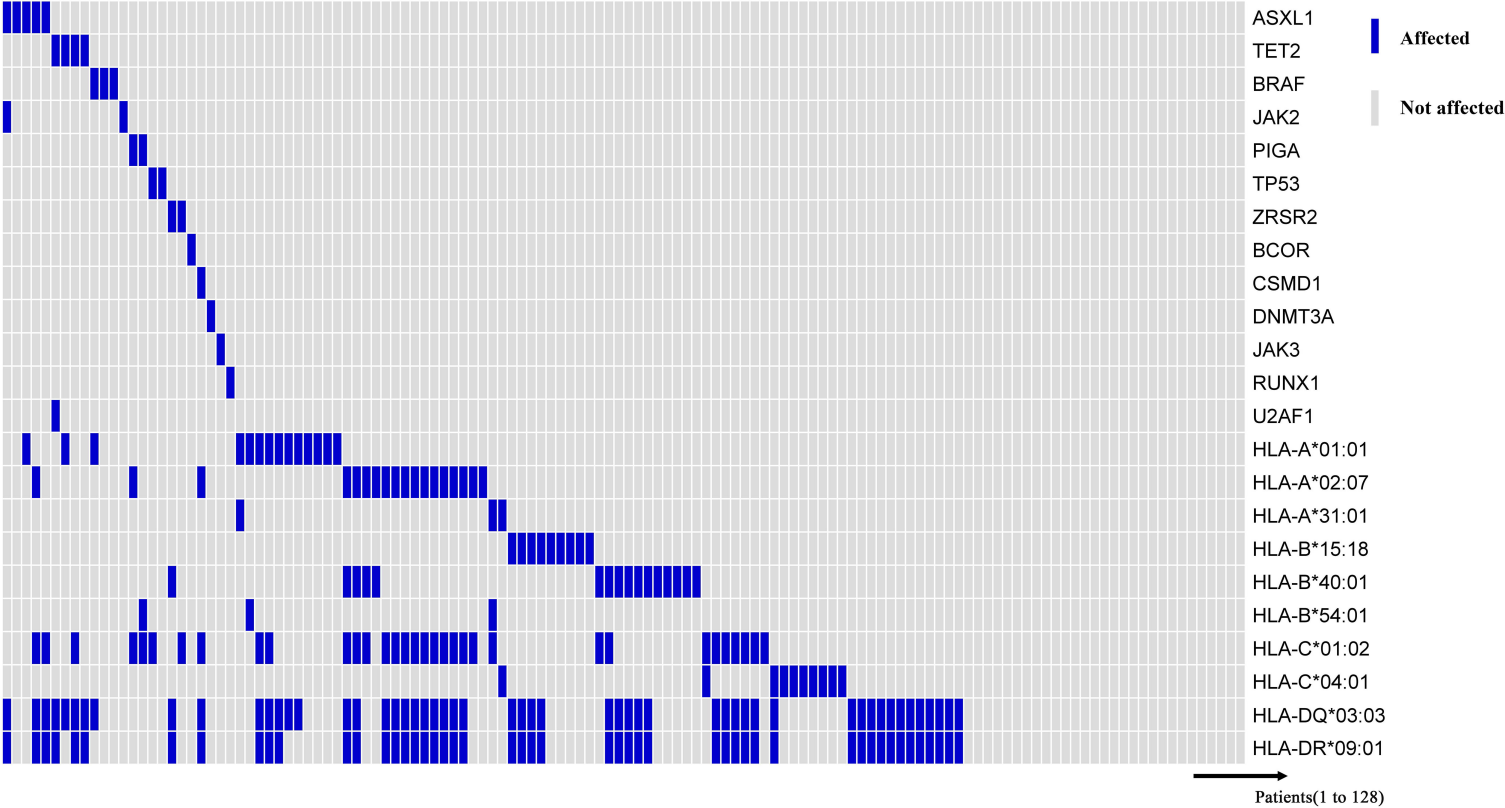
Somatic mutations associated with myeloid malignancies. Information on HLA alleles and somatic mutations at initial diagnosis of 128 patients. The horizontal axis represents each patient, and the vertical axis represents whether there is a mutation or with/without the HLA allele.

Previous studies have shown that PIGA, BCOR/BCORL1, and TET2 mutations were associated with better response to IST and better overall and progression-free survival, while ASXL1, BRAF, JAK2, TP53, ZRSR2, CSMD1, DNMT3A, JAK3, RUNX1, and U2AF1 mutations were associated with poor IST response and high-risk clonal evolution (25–27). Therefore, we compared the previously described HLA alleles associated with response to IST and clonal evolution with “unfavorable” and “favorable” somatic mutations (**Figure 4**). And, the results showed that these HLA alleles were not associated with “favorable” and “unfavorable” somatic mutations at initial diagnosis.

## 4 Discussion

AA is a bone marrow hematopoietic failure syndrome induced by various factors, especially the immune etiology. The HLA molecules play an important role in immune regulation by presenting endogenous and exogenous antigenic peptides to activate T helper cells. Previous studies have shown that CTLs recognize the auto-antigens presented on HSPCs through class I HLA molecules (28–30). And, HLA has been reported to be associated with disease susceptibility and the response to IST in AA patients (6–9). With the advance of genomic analysis techniques such as single nucleotide polymorphism (SNP) analysis, it was found that the loss of specific HLA alleles underlay the HLA-mediated immune regulation, which helped HSPCs to evade CTL-driven autoimmune attack (11, 31, 32). Given this, we retrospectively analyzed the HLA genotypes of AA patients, and tried to clarify their potential role in the basal clinical characteristics, the response to IST, clonal evolution, and long-term survival. Furthermore, by next-generation sequence, we attempted to uncover the possible molecular mechanism hidden in specific HLA genotypes.

Early prediction of IST response and knowing which patients may benefit from IST is essential. Previous studies have focused on HLA-II molecules, especially the HLA-DR15 allele. The relationship between HLA-DR15 and IST efficacy is controversial (7, 9, 33). Our studies showed that patients with the HLA-DR*15:01 allele were more susceptible to SAA, consistent with our previous findings (6), but not associated with the response to IST. In addition, our data revealed the connection between HLA-I molecules and the response to IST. Three HLA alleles, HLA-B*15:18, HLA-C*04:01, and HLA-A*31:01, were found to be associated with a superior response to IST. On the contrary, patients carrying HLA-A*02:07 and HLA-B*40:01 alleles had a poor response to IST. By comparing responses to IST at 3, 6, and 12 months, results implied that for patients with HLA-B*15:18 and HLA-C*04:01 alleles, most patients could respond in a short period after IST, and some patients with short-term non-response could respond within 12 months after IST. In contrast, the majority of patients carrying HLA-A*02:07 and HLA-B*40:01 alleles failed to respond to IST at an early period. And many of these patients who did not respond at 3 or 6 months after IST still had no response after long-term follow-up. Therefore, it is suggested that patients with HLA-A*02:07 and HLA-B*40:01 alleles who didn’t respond to IST at 3 or 6 months should consider salvage treatment as early as possible. Patients with the HLA-A*31:01 allele responded well to IST. Unfortunately, one patient with HLA-A*31:01 allele progressed to MDS at 32 months after IST. The clonal evolution of this case might contribute to the HLA-DQ*03:03 and HLA-DR*09:01 alleles carried by the patient, and the older age (44 years old). Previously, we described that the old patients with HLA-DQ*03:03 and HLA-DR*09:01 were prone to clonal evolution. Therefore, a more robust scoring system, including HLA-I alleles, HLA-II alleles and patient’s baseline clinical features, is needed to better predict the response to IST, thus providing more accurate individualized treatment options for AA patients.

The mechanism of how the response to IST depends on the HLA alleles is unclear. Past researches (12, 32) have suggested that some specific HLA alleles play a critical role in AA and may have a greater ability to present pathogenic autoantigens, or presented autoantigen may be the one that elicits T cell responses to HSPCs antigens. And these specific HLA alleles were prone to loss of expression *via* 6pLOH or somatic mutations to escape the attack of CTLs (12, 32), which further supported this notion. The proportion of these specific HLA alleles in AA patients was significantly higher than in healthy controls due to the pathogenic significance of these HLA alleles. Our previous study (6) showed that compared with healthy controls, the gene frequencies of HLA-A*02:07 and HLA-B*40:01 were significantly lower in AA patients. Besides, compared with healthy controls, HLA-B*40:01 gene frequency was significantly lower in VSAA patients, while HLA-A*02:07 was substantially lower in SAA patients (6). Interestingly, our present study showed the poor response of HLA-A*02:07 and HLA-B*40:01 to IST, including subgroup analysis results of VSAA and SAA groups. So, we inferred that the HLA allele with a higher frequency in AA than in healthy controls may have immune-related pathogenesis, so patients with this allele have a good response to IST. On the opposite, HLA alleles with lower susceptibility in AA may have a weak ability to present autoantigens and may exist other pathogenic mechanisms, so patients with the HLA allele respond unsatisfactorily to IST. However, our study was a retrospective analysis only, further studies were needed to confirm this point.

Several studies have confirmed that HLA alleles were associated with the high-risk clonal evolution. HLA-B*40:02 allele was identified to be prone to high-risk clonal evolution in Japanese (32), While, in four major ethnic groups in the United States, the corresponding allele was HLA-B*14:02 (13). Consistent with the results of Babushok (12), due to racial differences, our results indicated that the frequencies of the HLA-B*40:02 allele and the HLA-B*14:02 allele were low in Chinese population. Our results identified other two HLA alleles, HLA-A*01:01 and HLA-B*54:01, were associated with high-risk clonal evolution. Notably, patients carrying the HLA-A*01:01 allele have a more severe disease severity. As our previous study (23) found that age, disease severity, and cumulative days of treatment with recombinant human granulocyte colony-stimulating factor (rhuG-CSF) were risk factors for the evolution of AA into MDS/AML, we performed subgroup analysis. Our results proved that patients with HLA-A*01:01 and HLA-B*54:01 alleles still had a higher incidence of high-risk clonal evolution, regardless of age and disease severity, suggesting that the HLA allele is an independent predictor of high-risk clonal evolution. Therefore, for patients with HLA-A*01:01 and HLA-B*54:01 genotypes, it is necessary to closely monitor the clonal evolution after IST, to prospectively make clinically relevant treatment decisions and improve the long-term prognosis of patients.

When all patients were analyzed, no link between HLA alleles and long-term survival was found. However, through subgroup analysis, results showed that for patients aged ≥40 years and carrying both HLA-DQ*03:03 and HLA-DR*09:01 alleles, the long-term efficacy and long-term survival rate were significantly worse than those of non-carriers due to the high incidence of high-risk clonal evolution. In contrast, in patients aged<40 years, these two HLA alleles were more frequent but did not increase the risk of high-risk clonal evolution. Therefore, for patients with HLA-DQ*03:03 and HLA-DR*09:01 alleles and ages≥40 years old, early allogeneic hematopoietic stem cell transplantation may be considered instead of the routinely recommended IST therapy. However, our data is limited, we need to increase the dataset in further study.

In addition, to further explore the possible mechanism linking HLA alleles and outcomes after IST, we used the next-generation sequencing technology to analyze whether genetic mutations could partly uncover the mechanism behind it. Several somatic mutations related to the above HLA alleles were found in our study. CUX1 has been identified as a critical tumor suppressor gene located within a commonly deleted segment of chromosome arm 7q. CUX1 inactivation may be an early event similar to -7/del(7q), and clinical data strongly implicate CUX1 inactivation in myeloid disease development (34). However, our results showed that the HLA15:18 allele associated with CUX1 was associated with a good response to IST and was not associated with high-risk clonal evolution. The significance of other somatic mutations in AA was unclear. Then, our further comparisons revealed that the above HLA alleles were not associated with reported “favorable” and “unfavorable” somatic mutations in AA (25–27). These results may be due to the relatively small number of patients. Our study has limitations, and further studies are needed to expand the population to explore the underlying mechanisms.

In summary, our study revealed the relationship between specific HLA alleles and the outcome of IST in the Chinese population, identified unique HLA alleles associated with the high-risk clonal evolution, and performed subgroup analysis to explore the optimal individualized treatment options. In conclusion, HLA typing can help predict the response of AA patients to immunosuppressive therapy and the risk of high-risk clonal evolution, thereby helping to make more rational treatment decisions and improve the prognosis of AA patients.

## Data availability statement

The datasets for this article are not publicly available due to concerns regarding participant/patient anonymity. Requests to access the datasets should be directed to the corresponding author.

## Ethics statement

The studies involving human participants were reviewed and approved by the ethics committee of Institute of Hematology and Blood Diseases Hospital, Chinese Academy of Medical Sciences. Written informed consent to participate in this study was provided by the participants’ legal guardian/next of kin.

## Author contributions

LC, MG, and YZ designed the research and drafted the manuscript. JH, XR, YS, XL, JH, MW, NN, JZ, JP helped with collection and assembly clinical data. All authors contributed to the article and approved the submitted version.
