# Supplementary material for: Association between human leukocyte antigen and immunosuppressive treatment outcomes in Chinese patients with aplastic anemia
Source: Front Immunol. 2023 Jan 30;14:1056381. doi: 10.3389/fimmu.2023.1056381 (PMC9923019; doi:10.3389/fimmu.2023.1056381)
Supplement: Supplementary file 1 [file Table_1.docx]

Supplementary Material

# Supplementary Tables

Supplemental Table 1. HLA alleles associated with clinical parameters

| HLA allele | Total^a^ | Male sex  n(%)^b^ | P value | OR(95%CI) | SAA  n(%)^c^ | P value | OR(95%CI) | PNH clone  n(%)^d^ | P value | OR(95%CI) |
| --- | --- | --- | --- | --- | --- | --- | --- | --- | --- | --- |
| A*01:01 | 19 | 13(10.7%) | 0.426 | 1.505(0.547-4.316) | 5(5.2%) | **0.047** | 0.353(0.122-1.021) | 2(6.3%) | 0.744 | 0.600(0.132-2.734) |
| B*13:01 | 26 | 17(14%) | 0.541 | 1.308(0.552-3.097) | 10(10.3%) | 0.296 | 0.639(0.275-1.486) | 4(12.5%) | 1.000 | 0.961(0.308-3.003) |
| B*15:01 | 21 | 13(10.7%) | 0.843 | 1.098(0.434-2.782) | 10(10.3%) | 0.969 | 0.982(0.397-2.427) | 5(15.6%) | 0.340 | 1.782(0.603-5.271) |
| B*40:02 | 17 | 10(8.3%) | 0.925 | 0.952(0.347-2.614) | 6(6.2%) | 0.272 | 0.563(0.200-1.587) | 2(6.3%) | 1.000 | 0.689(0.150-3.170) |
| B*44:03 | 14 | 10(8.3%) | 0.362 | 1.734(0.525-5.731) | 9(9.3%) | 0.207 | 2.045(0.661-6.333) | 6(18.8%) | **0.012** | 4.673(1.500-14.562) |
| B*46:01 | 39 | 27(22.3%) | 0.186 | 1.652(0.782-3.488) | 14(14.4%) | 0.092 | 0.540(0.262-1.112) | 7(21.9%) | 0.688 | 1.208(0.480-3.036) |
| C*08:01 | 33 | 14(11.6%) | **0.025** | 0.427(0.200-0.912) | 17(17.5%) | 0.660 | 1.182(0.560-2.494) | 4(12.5%) | 0.522 | 0.695(0.226-2.132) |
| DQ*03:01 | 72 | 46(38%) | 0.389 | 1.297(0.717-2.349) | 40(41.2%) | 0.111 | 1.601(0.897-2.858) | 11(34.4%) | 0.870 | 0.936(0.423-2.071) |
| DQ*0303 | 78 | 47(38.8%) | 0.935 | 1.024(0.575-1.826) | 27(27.8%) | **0.002** | 0.408(0.227-0.734) | 10(31.3%) | 0.351 | 0.682(0.304-1.530) |
| DQ*06:01 | 37 | 21(17.4%) | 0.666 | 0.853(0.415-1.755) | 18(18.6%) | 0.932 | 1.031(0.505-2.105) | 6(18.8%) | 0.945 | 1.035(0.393-2.728) |
| DQ*06:02 | 58 | 29(24%) | 0.068 | 0.565(0.305-1.047) | 34(35.1%) | 0.056 | 1.821(0.982-3.378) | 15(46.9%) | **0.013** | 2.606(1.200-5.660) |
| DR*08:03 | 19 | 14(11.6%) | 0.198 | 1.989(0.687-5.755) | 7(7.2%) | 0.306 | 0.603(0.227-1.600) | 3(9.4%) | 1.000 | 0.996(0.273-3.637) |
| DR*09:01 | 74 | 43(35.5%) | 0.693 | 0.889(0.497-1.592) | 27(27.8%) | **0.013** | 0.476(0.265-0.857) | 10(31.3%) | 0.491 | 0.753(0.335-1.691) |
| DR*15:01 | 64 | 31(25.6%) | **0.024** | 0.501(0.274-0.915) | 40(41.2%) | **0.005** | 2.368(1.288-4.355) | 15(46.9%) | **0.044** | 2.179(1.009-4.704) |
| All patients | 202 | 121(59.9%) |  |  | 97(48%) |  |  | 32(15.8%) |  |  |

^a^Total number of people carrying the HLA allele; ^b^% of total male patients; ^c^%of total SAA patients; ^d^%of total PNH clone positive patients; P-values are results of the comparison between patients carrying the HLA allele and other patients not carrying the HLA allele; SAA, severe aplastic anemia; PNH, Paroxysmal nocturnal hemoglobinuria; ANC, Neutrophil count; PLT, Platelet count; ARC, Reticulocyte count; ALC, absolute lymphocyte count. HLA alleles ≥ 10 are presented in the table.

Supplemental Table 1. HLA alleles associated with clinical parameters (continued)

| HLA allele | Total | ANC, ×10^9^/L  Median(Range) | P value | PLT, ×10^9^/L  Median(Range) | P value | ARC, ×10^9^/L  Median(Range) | P value | ALC, ×10^9^/L  Median(Range) | P value | Age, y  Median(  Range) | P value |
| --- | --- | --- | --- | --- | --- | --- | --- | --- | --- | --- | --- |
| A*01:01 | 19 | 0.18(0.02-0.71) | 0.360 | 8(2-46) | 0.957 | 12.7(1.6-65.0) | 0.720 | 1.63(0.19-2.10) | 0.787 | 18(10-56) | 0.554 |
| B*13:01 | 26 | 0.16(0.00-1.11) | 0.695 | 8.5(2.0-19.0) | 0.808 | 8.3(2.9-65.0) | 0.396 | 1.06(0.03-3.02) | 0.116 | 26(7-60) | **0.048** |
| B*15:01 | 21 | 0.34(0.00-1.20) | 0.452 | 5(1-17) | **0.001** | 12(2.3-71.5) | 0.802 | 1.13(0.43-7.81) | 0.601 | 31(5-54) | 0.053 |
| B*40:02 | 17 | 0.09(0.00-0.63) | 0.028 | 8(1-25) | 0.605 | 7.1(1.9-23.8) | 0.058 | 1.33(0.24-2.66) | 0.986 | 20(7-53) | 0.650 |
| B*44:03 | 14 | 0.265(0.050-0.920) | 0.701 | 9.5(2.0-34.0) | 0.738 | 12.05(3.60-71.50) | 0.687 | 1.665(0.580-3.430) | 0.197 | 26.5(10-47) | 0.719 |
| B*46:01 | 39 | 0.16(0.01-1.09) | 0.066 | 8(1-22) | 0.441 | 11.8(2.3-82.1) | 0.483 | 1.27(0.11-7.62) | 0.804 | 22(8-48) | 0.206 |
| C*08:01 | 33 | 0.19(0.00-1.12) | 0.911 | 11(3-25) | 0.167 | 10(1.6-44.7) | 0.292 | 1.4(0.29-4.55) | 0.249 | 24(10-60) | 0.570 |
| DQ*03:01 | 72 | 0.3(0.00-1.31) | **0.039** | 9(2-25) | 0.759 | 10.7(1.6-83.3) | 0.362 | 1.32(0.03-3.02) | 0.684 | 26(6-60) | **0.007** |
| DQ*03:03 | 78 | 0.165(0.000-1.120) | **0.023** | 8.5(1.0-46.0) | 0.183 | 9.95(1.90-82.10) | 0.261 | 1.245(0.110-7.810) | 0.798 | 22(5-57) | **0.037** |
| DQ*06:01 | 37 | 0.2(0.00-1.31) | 0.959 | 10(1-28) | 0.741 | 9.2(2.3-83.3) | 0.571 | 1.36(0.30-7.81) | 0.415 | 17(5-57) | **0.017** |
| DQ*06:02 | 58 | 0.29(0.00-1.21) | 0.317 | 10(2-34) | 0.478 | 11.6(1.9-44.7) | 0.826 | 1.385(0.030-4.550) | 0.461 | 23.5(6.0-60.0) | 0.250 |
| DR*08:03 | 19 | 0.16(0.00-1.31) | 0.771 | 11(1-28) | 0.537 | 11.8(2.3-83.3) | 0.569 | 1.32(0.30-7.81) | 0.822 | 15(5-41) | **0.001** |
| DR*09:01 | 74 | 0.17(0.00-1.12) | **0.031** | 8.5(1.0-34.0) | 0.099 | 9.95(1.90-82.10) | 0.241 | 1.245(0.110-7.810) | 0.950 | 22(5-57) | **0.02** |
| DR*15:01 | 64 | 0.335(0.000-1.210) | 0.055 | 10(2-34) | 0.422 | 11.6(1.9-44.7) | 0.955 | 1.405(0.030-4.550) | 0.239 | 24.5(6.0-60.0) | 0.272 |
| All patients | 202 | 0.24(0.00-1.31) |  | 9(1-46) |  | 11.15(1.60-83.30) |  | 1.315(0.000-7.810) |  | 23(5-60) |  |

^a^Total number of people carrying the HLA allele; ^b^% of total male patients; ^c^%of total SAA patients; ^d^%of total PNH clone positive patients; P-values are results of the comparison between patients carrying the HLA allele and other patients not carrying the HLA allele; SAA, severe aplastic anemia; PNH, Paroxysmal nocturnal hemoglobinuria; ANC, Neutrophil count; PLT, Platelet count; ARC, Reticulocyte count; ALC, absolute lymphocyte count. HLA alleles ≥ 10 are presented in the table.

Supplemental Table 2. Phenotype of frequencies of HLA alleles according to Long-term response to immunosuppressive therapy or incidence of high-risk clonal evolution

| HLA allele | Total | Long-term response to immunosuppressive therapy | | | High-risk clonal evolution | | |
| --- | --- | --- | --- | --- | --- | --- | --- |
|  |  | Response(n=60)  n(%) | Nonresponse(n=35)  n(%) | P value | Yes(n=11)  n(%) | No(n=84)  n(%) | P value |
| HLA-A |  |  |  |  |  |  |  |
| A*01:01 | 7 | 2(3.3) | 5(14.3) | 0.096 | 3(27.2) | 4(4.8) | **0.032** |
| A*02:01 | 32 | 20(33.3) | 12(34.3) | 0.925 | 2(18.2) | 30(35.7) | 0.324 |
| A*02:06 | 23 | 15(25.0) | 8(22.9) | 0.814 | 1(9.1) | 22(26.2) | 0.286 |
| A*02:07 | 17 | 8(13.3) | 9(25.7) | 0.129 | 4(36.4) | 13(15.5) | 0.105 |
| A*03:01 | 5 | 2(3.3) | 3(8.6) | 0.354 | 1(9.1) | 4(4.8) | 0.467 |
| A*11:01 | 27 | 18(30) | 9(25.7) | 0.655 | 5(45.5) | 22(26.2) | 0.284 |
| A*24:02 | 26 | 19(31.7) | 7(20.0) | 0.219 | 1(9.1) | 25(29.8) | 0.279 |
| A*30:01 | 6 | 5(8.3) | 1(2.9) | 0.408 | 0(0) | 6(7.1) | 1.000 |
| A*31:01 | 8 | 7(11.7) | 1(2.9) | 0.251 | 1(9.1) | 7(8.3) | 1.000 |
| A*33:03 | 11 | 7(11.7) | 4(11.4) | 1.000 | 0(0) | 11(13.1) | 0.352 |
| HLA-B |  |  |  |  |  |  |  |
| B*07:02 | 5 | 5(8.3) | 0(0) | 0.154 | 0(0) | 5(6.0) | 1.000 |
| B*13:01 | 16 | 10(16.7) | 6(17.1) | 1.000 | 3(27.2) | 13(15.5) | 0.388 |
| B*13:02 | 7 | 6(10.0) | 1(2.9) | 0.255 | 0(0) | 7(8.3) | 1.000 |
| B*15:01 | 10 | 6(10.0) | 4(11.4) | 1.000 | 0(0) | 10(11.9) | 0.599 |
| B*15:11 | 5 | 3(5.0) | 2(5.7) | 1.000 | 0(0) | 5(6.0) | 1.000 |
| B*15:18 | 8 | 8(13.3) | 0(0) | **0.025** | 0(0) | 8(9.5) | 0.590 |
| B*35:01 | 5 | 4(6.7) | 1(2.9) | 0.649 | 0(0) | 5(6.0) | 1.000 |
| B*40:01 | 16 | 6(10.0) | 10(28.6) | **0.020** | 4(36.4) | 12(14.3) | 0.086 |
| B*40:02 | 5 | 5(8.3) | 0(0) | 0.154 | 0(0) | 5(6.0) | 1.000 |
| B*40:06 | 8 | 5(8.3) | 3(8.6) | 1.000 | 1(9.1) | 7(8.3) | 1.000 |
| B*44:03 | 7 | 6(10.0) | 1(2.9) | 0.255 | 0(0) | 7(8.3) | 1.000 |
| B*46:01 | 21 | 13(21.7) | 8(22.9) | 0.893 | 5(45.5) | 16(19.0) | 0.061 |
| B*48:01 | 10 | 8(13.3) | 2(5.7) | 0.315 | 0(0) | 10(11.9) | 0.599 |
| B*51:01 | 10 | 5(8.3) | 5(14.3) | 0.490 | 1(9.1) | 9(10.7) | 1.000 |
| B*54:01 | 5 | 1(1.7) | 4(11.4) | 0.060 | 3(27.2) | 2(2.4) | **0.010** |
| B*58:01 | 5 | 2(3.3) | 3(8.6) | 0.354 | 0(0) | 5(6.0) | 1.000 |
| HLA-C |  |  |  |  |  |  |  |
| C*01:02 | 23 | 14(23.3) | 9(25.7) | 0.794 | 5(45.5) | 18(21.4) | 0.128 |
| C*03:02 | 5 | 2(3.3) | 3(8.6) | 0.354 | 0(0) | 5(6.0) | 1.000 |
| C*03:03 | 12 | 8(13.3) | 4(11.4) | 1.000 | 0(0) | 12(14.3) | 0.348 |
| C*03:04 | 24 | 14(23.3) | 10(28.6) | 0.571 | 5(45.5) | 19(22.6) | 0.138 |
| C*04:01 | 13 | 12(20.0) | 1(2.9) | **0.027** | 0(0) | 13(15.5) | 0.351 |
| C*06:02 | 12 | 8(13.3) | 4(11.4) | 1.000 | 2(18.2) | 10(11.9) | 0.626 |
| C*07:02 | 21 | 15(25.0) | 6(17.1) | 0.373 | 0(0) | 21(25.0) | 0.115 |
| C*07:04 | 5 | 4(6.7) | 1(2.9) | 0.649 | 1(9.1) | 4(4.8) | 0.467 |
| C*08:01 | 20 | 11(18.3) | 9(25.7) | 0.395 | 3(27.3) | 17(20.2) | 0.694 |
| C*08:22 | 5 | 5(8.3) | 0(0) | 0.154 | 0(0) | 5(6.0) | 1.000 |
| C*14:02 | 5 | 3(5.0) | 2(5.7) | 1.000 | 0(0) | 5(6.0) | 1.000 |
| C*15:02 | 10 | 5(8.3) | 5(14.3) | 0.490 | 2(18.2) | 8(9.5) | 0.326 |
| HLA-DQB1 |  |  |  |  |  |  |  |
| DQ*02:01 | 6 | 2(3.3) | 4(11.4) | 0.189 | 0(0) | 6(7.1) | 1.000 |
| DQ*02:02 | 11 | 9(15.0) | 2(5.7) | 0.205 | 0(0) | 11(13.1) | 0.352 |
| DQ*03:01 | 28 | 18(30.0) | 10(28.6) | 0.883 | 3(27.3) | 25(29.8) | 1.000 |
| DQ*03:02 | 8 | 7(11.7) | 1(2.9) | 0.251 | 0(0) | 8(9.5) | 0.590 |
| DQ*03:03 | 35 | 21(35.0) | 14(40.0) | 0.626 | 7(63.6) | 28(33.3) | 0.092 |
| DQ*04:01 | 13 | 9(15.0) | 4(11.4) | 0.762 | 2(18.2) | 11(13.1) | 0.644 |
| DQ*06:01 | 17 | 11(18.3) | 6(17.1) | 0.884 | 1(9.1) | 16(19.0) | 0.682 |
| DQ*06:02 | 30 | 18(30.0) | 12(34.3) | 0.665 | 2(18.2) | 28(33.3) | 0.493 |
| HLA-DRB1 |  |  |  |  |  |  |  |
| DR*03:01 | 8 | 3(5.0) | 5(14.3) | 0.140 | 1(9.1) | 7(8.3) | 1.000 |
| DR*04:05 | 13 | 9(15.0) | 4(11.4) | 0.762 | 2(18.2) | 11(13.1) | 0.644 |
| DR*07:01 | 11 | 9(15.0) | 2(5.7) | 0.205 | 0(0) | 11(13.1) | 0.352 |
| DR*08:03 | 7 | 6(10.0) | 1(2.9) | 0.255 | 0(0) | 7(8.3) | 1.000 |
| DR*09:01 | 35 | 22(37.7) | 13(37.1) | 0.963 | 6(54.5) | 29(34.5) | 0.318 |
| DR*11:01 | 7 | 5(8.3) | 2(5.7) | 1.000 | 1(9.1) | 6(7.1) | 0.590 |
| DR*12:01 | 6 | 3(5.0) | 3(8.6) | 0.666 | 0(0) | 6(7.1) | 1.000 |
| DR*12:02 | 11 | 7(11.4) | 4(11.4) | 1.000 | 1(9.1) | 10(11.9) | 1.000 |
| DR*15:01 | 33 | 19(31.7) | 14(40.0) | 0.441 | 3(27.3) | 30(35.7) | 0.742 |
| DR*15:02 | 7 | 3(5.0) | 4(11.4) | 0.417 | 0(0) | 7(8.3) | 1.000 |

HLA alleles ≥ 5 are presented in the table
